# Humor Styles and Empathy in Junior-School Children

**DOI:** 10.5964/ejop.v16i1.1934

**Published:** 2020-03-03

**Authors:** Caitlin Charlotte Halfpenny, Lucy Amelia James

**Affiliations:** a School of Psychology, Keele University, Keele, United Kingdom; Department of Psychology and Counselling, Webster University Geneva, Geneva, Switzerland

**Keywords:** children, humor, humor styles, empathy, quantitative research

## Abstract

Humor is a complex phenomenon. For one individual a joke may be perceived as comical, yet for another, the same joke may be deemed completely inappropriate. The appropriate use of humor is perhaps dependent on how a humorist relates to, understands and can empathize with their audience. Thus, the present research aimed to determine whether empathy is related to junior-school children’s use of different humor styles. It has been proposed that four styles of humor exist, two of which are thought to be adaptive (affiliative and self-enhancing) and two of which are thought to be maladaptive (aggressive and self-defeating). However, research exploring the role of humor styles in younger children’s development has been limited. To investigate this the Humor Styles Questionnaire for young children (HSQ-Y) and the Thinking and Feeling Questionnaire were administered to 214 UK children aged 9-11 years old. Correlational analyses revealed that self-enhancing humor is associated with cognitive empathy, affective empathy and sympathy, affiliative humor is positively associated with cognitive empathy specifically and aggressive humor is negatively associated with affective empathy and sympathy. Possible explanations for these associations are explored, with a consideration of the direction for future research in this predominantly unexplored field of study.

## The Four Humor Styles Approach

Humor is a universal experience, occurring many times in everyday life, in all cultures across the globe (Frew, 2006). Martin, Puhlik-Doris, Larsen, Gray, and Weir (2003) aimed to develop a multidimensional measure of humor that incorporated adaptive and maladaptive styles of humor, in addition to both self and other directed functions that are not tapped by other measures. Martin et al. (2003) proposed that humor could be categorized into four distinct styles that could be measured. Subsequently, the Humor Styles Questionnaire (HSQ) was developed, measuring affiliative, self-enhancing, aggressive and self-defeating humor.

Martin et al. (2003) described affiliative humor as a tendency to say funny things and to make jokes to facilitate social interaction through the amusement of others. Affiliative humor is adaptive as it is positively correlated with positive social interaction (Norrick & Chiaro, 2009), social intimacy, self-esteem and overall psychological wellbeing and negatively correlated with depression and anxiety (Martin et al., 2003). Self-enhancing humor is also adaptive, although it is self-directed. Thought to be closely related to the concept of coping humor and consistent with the definition of humor as a healthy defence mechanism (Freud, 1928), self-enhancing humor can be used to release tension and boost mood to overcome hardship. It is positively correlated to overall psychological wellbeing, optimism, self-esteem, and satisfaction with social support and negatively related to anxiety and depression (Martin et al., 2003).

In contrast, aggressive humor is described as the use of jokes, teasing or sarcasm to enhance the self at the expense of others, regardless of the potentially hurtful impact. Akin to Zillmann’s (1983) description of humor which proposed that the intent of humor was to target others to gain superiority over them. Accordingly, Martin et al. (2003) reported that aggressive humor is maladaptive as it is positively related to hostility and negatively correlated to self-reported agreeableness and conscientiousness. As such the use of this humor style precludes prosocial behaviour and has negative effects on one’s social relationships and personal wellbeing. Finally, self-defeating humor which refers to an individual’s attempts to make others laugh at the expense of the self (Martin et al., 2003). This humor style, largely untapped by previous humor scales has been found to be positively correlated with bad mood, hostility, self-reported anxiety and depression, and negatively related to overall psychological wellbeing, self-esteem and satisfaction with social support (Martin et al., 2003). It is predominantly maladaptive in nature as it is thought to reflect a social deficiency, emotional neediness and low self-esteem (Fabrizi & Pollio, 1987) that inhibits healthy social relationships and psychological wellbeing.

The HSQ has been found to exhibit good psychometric properties across a range of cultures (Chen & Martin, 2007; Kazarian & Martin, 2004; Saroglou & Scariot, 2002). Martin et al. (2003) stressed that though the four humor styles are distinguishable, there should be some overlap between them. For instance, self-defeating and self-enhancing humor should be related to a degree, as they are both self-directed, however correlation results of the four humor styles did not support this relationship (Martin et al., 2003).

## Applying the Humor Styles Approach to Children

Klein and Kuiper (2006) praised the humor styles approach for considering individual differences in adaptive and maladaptive forms of humor. However, they highlighted that this model had not been considered in respect to children’s humor. Based on evidence that humor is fundamental to children’s social interactions (Goleman, 2006), Klein and Kuiper (2006) proposed that the humor styles model could be applied to children, particularly during middle childhood.

In line with previous associations between children’s humor and their status within the peer group (Masten, 1986; Sherman, 1988), Klein and Kuiper (2006) proposed that specific humor styles could influence children’s peer relationships and have important implications for their social competence and adjustment. As such, they suggested that children’s use of adaptive humor has a cyclical effect on social competence; with the use of these humor styles, opportunities to develop and understand positive uses of humor increases, thus allowing the growth of these humor styles and contributing overall to positive social development. Similarly, use of maladaptive humor styles could result in a vicious cycle of peer rejection, in which peer rejection deprives children of the opportunity to develop more adaptive humor styles. Overall, Klein and Kuiper’s (2006) proposals are consistent with Martin et al.’s (2003) original humor styles model, providing an extension by considering adaptive and maladaptive humor in middle childhood.

Erickson and Feldstein (2007) attempted to utilize the adult HSQ with adolescents, however, they found unacceptable reliability coefficients for the maladaptive humor styles. As the adult-standardized HSQ could not be applied as a psychometrically sound instrument for the measure of adolescents’ humor styles, Fox, Dean, and Lyford (2013) adapted it to create the child HSQ. Although this measure was found to have test-retest reliability across two time points, confirmatory factor analyses suggested that the four factor structure was not appropriate for children below the age of 11 years old. It appeared that the self-directed humor styles were not present for this younger age group, thus Fox et al. (2013) questioned whether young children could sufficiently self-report their own humor use or if self-directed forms of humor emerged later in life with cognitive development. As such, it was recommended that a two factor questionnaire comprised of affiliative and aggressive humor would better represent younger populations’ use of humor. However, an interview study by James and Fox (2016a) indicated that although individual differences were evident, some children aged 8-11 years old were able to discuss the use of self-enhancing and self-defeating humor. It appeared that a HSQ for younger children should incorporate all four styles of humor.

Consequently, James and Fox (2016b) successfully developed a four factor humor styles questionnaire for younger children (HSQ-Y), aged 8-11 years old. Children’s self-reported use of adaptive humor styles were found to be positively associated with self-perceived social competence and peer acceptance and negatively correlated with loneliness. These findings were supportive of Martin et al.’s (2003) original findings with young adults; adaptive humor styles were positively related to overall psychological wellbeing and social outcomes. Moreover, James and Fox (2016b) found a low positive relationship between self-enhancing and self-defeating humor among the correlations of the four humor styles, as supposed but not supported by Martin et al. (2003). James and Fox (2016b) also demonstrated the validity of the HSQ-Y by utilizing peer reports to corroborate children’s self report and by administering the measures on two occasions to achieve test re-test reliability. The application of the four humor styles approach to children was further validated by James and Fox (2018) who established longitudinal relationships between children’s self-reported humor on the HSQ-Y and psychological adjustment. Overall, these findings reinforced the justification for applying Martin et al.’s (2003) HSQ to children, providing an opportunity for the present research to utilize the HSQ-Y in the exploration of children’s humor styles in relation to empathy.

## Empathy

One generally accepted definition of empathy is the apprehension and sharing of perceived emotion with another (Eisenberg & Strayer, 1990): this definition is brief and exceedingly broad. One of the key controversies in the definitional issues of empathy is whether it is a cognitive or affective phenomenon (Ickes, 1997). While the earliest studies considered cognitive and affective components of empathy as separate (Duan & Hill, 1996; Kerr & Speroff, 1954), most contemporary research incorporates both components, using multidimensional measures such as the Interpersonal Reactivity Index (IRI; Davis, 1980) which avoids artificial compartmentalization of cognitive and affective empathy by studying these elements in parallel (Staub, 1987). Though the IRI has good psychometric properties when used with adults (Carey, Fox, & Spraggins, 1988; Hawk, Keijsers, Branje, Van der Graaff, de Wied, & Meeus, 2013), it is not suitable for use with children. Though Garton and Gringart (2005) devised a successful adaptation of the IRI for use with children aged 8-11 years old, it has been criticized profoundly for containing a number of items that equate empathy with sympathy (Fernández, Dufey, & Kramp, 2011).

Although sympathy can be considered related to empathy, it is important for researchers to distinguish critical differences between them. For example, Eisenberg and Strayer (1990) differentiated sympathy as having an additional response to empathy. An individual may have empathy for another but not necessarily sympathy; it is possible for one to understand another’s feelings and have empathy for them but not have sympathy, perhaps because they feel that the other person deserves their distress. Although empathy and sympathy can be linked, it is important that measures do not equate these constructs, rather measure them as separate subscales (Villadangos, Errasti, Amigo, Jolliffe, & Garcia-Cueto, 2016).

Accordingly, the present research utilized an extension of Zoll and Enz’s (2005) Empathy Questionnaire (EQ). While the EQ has been established as an accurate measure of cognitive and affective empathy, with a validated two factor structure amongst children aged 8-14 years old from the UK, Portugal, Germany (Zoll & Enz, 2005) and Bolivia (Roth, 2018), the present research endeavored to incorporate a measure of sympathy. The extended version of the EQ, the Thinking and Feeling Questionnaire (Zoll & Enz, 2005) was utilized to measure cognitive and affective empathy, alongside sympathy.

### Cognitive Empathy

Cognitive empathy refers to the individual’s ability to intellectually assume the perspective of another person (Gladstein, 1983). Essentially Zoll and Enz (2005) characterized cognitive empathy as the thinking and understanding aspect of empathy, the “Theory of Mind” construct that Davis (1983) referred to as “perspective-taking”. It is the ability to represent the internal states of others by decoding their behaviours, labelling their emotions and using situational cues. From a developmental perspective, this type of empathy is dependent on the cognitive development of children and so, does not develop until children possess the relevant cognitive capabilities.

### Affective Empathy

Under Piagetian influence empathy has been equated with cognitive abilities such as perspective-taking until recently in which an affective component has been acknowledged (Lamb & Keller, 2019). According to Zoll and Enz (2005) affective empathy equated to Davis’ (1983) “empathic concern” in which an individual is able to feel with another person, experiencing a congruent emotion in response to their condition. Affective empathy can be considered a result of cognitive empathy or as an established expression of a child’s innate drive to respond appropriately to the emotions of others (Zoll & Enz, 2005). These opposing perspectives present a matter of contention within the literature; as the proposal that affective empathy is the result of the cognitive apprehension of another’s emotional state suggests that cognitive empathy is a prerequisite of the emotional component of empathy (Eisenberg, 2005). While Wispe (1987) argued that affective empathy cannot occur without the fundamental cognitive components that work to serve the experience of affective empathy, Shamay-Tsoory (2009) contended that affective empathy is entirely separate and represents the other-orientated prosocial behaviours displayed by infants from a young age.

It appeared that employing a measure that incorporated both affective and cognitive components of empathy and examining them in parallel was the most appropriate, particularly as the relationship between these components and their development in childhood remained a complex issue (Raine & Chen, 2018). The use of the Thinking and Feeling Questionnaire allowed for the examination of the possible interactive effects of affective and cognitive empathy.

### Sympathy

Zoll and Enz (2005) referred to sympathy as a construct related to, but separate from empathy. Sympathy is a vicarious emotional reaction and concern for another that is based on the understanding of their emotional condition. Sympathy not only includes empathizing but also entails positive regard and intention to help. Rather than feeling with another, sympathy is the sympathizers’ concern and subsequent action to help (Eisenberg & Miller, 1987).

Considering the shortcomings of the IRI for equating the measurement of sympathy with that of empathy, the use of the Thinking and Feeling Questionnaire will allow each of these constructs to be measured in their own right.

## Humor and Empathy

There are a substantial number of parallels between humor and empathy, such as the similarities in their emergence in child development. Both humor and empathy mature across cognitive development in the social context, though the foundational elements of each are often considered innate (Fogel et al., 1997; Sroufe & Wunsch, 1972). With cognitive development children’s innate sensitivity to humor and tendency to appreciate another’s emotions matures. Though due to the cognitive demand of both humor and empathy, the emergence of the most sophisticated forms of these qualities do not appear until later in development. For instance, at the age children become less egocentric as they begin to recognize that the thoughts and feelings of others may differ from their own (Piaget, 1970). It is with this cognitive capability that children are able to develop the most advanced level of social understanding, Theory of Mind (ToM). ToM is the recognition that the thoughts, feelings, beliefs, desires and intentions of another may differ from our own (Howe, 2013). It is this capacity to feel things from another person’s perspective that gives rise to more sophisticated, other-orientated forms of humor and is the basis of empathy. Finally, the social nature of humor and empathy relative to their function and impact on social behaviours and relationships are similar. Both humor and empathy are perceived as positive and desirable interpersonal qualities that promote a range of social functions across the lifespan. For instance, empathy is a valuable characteristic in the workplace (Arnold et al., 2016) and in romantic relationships (Perrone-McGovern et al., 2014), much like a “sense of humor” is highly valued in children, parents, employees, partners and friendships (Ziv, 2010). Overall, it appears that humor and empathy are intrinsically linked to prosocial outcomes and behaviour.

Despite the wealth of similarities between humor and empathy, few researchers have endeavored to explore the relationship between these variables. Martin and Dutrizac (2004) found that greater use of adaptive humor styles and lesser use of maladaptive humor styles among undergraduate students can result in more positive social interactions in daily life. Moreover, the giving and receiving of empathy was characteristically linked to the prosocial nature of adaptive humor styles. However, this study was limited by the use of a single, global measure of empathy. Martin and Dutrizac (2004) proposed that additional measures or multiple subscales of empathy could improve the validity of the findings. Accordingly, Hampes (2010) examined the associations between humor styles and empathy using the IRI, a multidimensional measure of empathy. Hampes (2010) found that adaptive humor styles were positively associated with empathic concern, sympathy, compassion and perspective-taking and aggressive humor was negatively associated with perspective-taking and empathic concern.

Beyond the observed associations between humor and empathy, Wu, Lin, and Chen (2016) proposed that differential relationships in humor styles are mediated by gender differences in empathy. Overall, Wu, Lin, and Chen (2016) findings strengthened those of Hampes (2010), substantiating the rationale for the exploration of associations between humor styles and empathy; not only is there an association between humor styles and empathy but an individual’s tendency to use either adaptive or maladaptive humor is mediated by gender differences in empathic skills and abilities. For instance, females were significantly more likely to score highly on perspective-taking empathy and consequently, were more likely to report using adaptive humor styles. Whereas males were more likely to score low on empathic concern and consequently, reported using maladaptive humor styles.

Despite the apparent similarities and associations between humor and empathy, few researchers (Hampes 2010; Martin & Dutrizac, 2004; Wu, Lin, & Chen, 2016) appear to have investigated the differential associations between specific humor styles and various measures of empathy. Moreover, these studies utilized the IRI (Davis, 1980) to measure empathy. As previously discussed, the IRI has been criticized for equating sympathy with the construct of empathy on the same subscale, therefore it was imperative that the present research implemented a measure such as the Thinking and Feeling Questionnaire (Zoll & Enz, 2005) which examined affective empathy, cognitive empathy and sympathy as related but separate constructs. Besides the aforementioned studies have been conducted with young adults and adolescents only, the evidence for associations between humor styles and empathy among children is absent.

## The Present Research

The primary aim of the present research was to bridge the gap in the study of children’s use of humor styles by exploring associations with cognitive empathy, affective empathy and sympathy. As the association between junior- school aged children’s use of humor styles and empathy were yet to be explored, the present research employed correlational analyses to establish the presence of any relationships. Based on previous research conducted with young adults it was hypothesized that: adaptive humor styles would be positively associated with empathy, maladaptive humor styles would be negatively correlated with empathy, girls would self-report using adaptive humor styles and empathy significantly more than boys and boys would self-report using maladaptive humor styles significantly more and empathy significantly less than girls.

## Method

### Participants

214 children were recruited from two primary schools in the North West of England. Children completed a demographic form for their age and gender. The sample consisted of year 5 and year 6 pupils aged 9-11 years with a mean age of 10.12 years (*SD* = 0.68). There were 97 boys and 116 girls. Parental consent was obtained using the opt-out method.

### Materials

A questionnaire was used to assess children’s humor styles and empathy. This questionnaire employed two self-report scales, the HSQ-Y (James & Fox, 2016b) and the Thinking and Feeling Questionnaire (Zoll & Enz, 2005).

The HSQ-Y is an adapted version of Martin et al.’s (2003) original HSQ that measures affiliative, self-enhancing, aggressive and self-defeating humor. However, the HSQ-Y was considered more appropriate for children with a Flesch reading ease score of 84.90 (US grade level = 3.60, UK year 4, age 8-9 years). This measure presented children with 24 questionnaire items in which each dimension of humor style was assessed on a subscale of 6 items. An example question from the affiliative humor subscale is: “My jokes and funny stories make people laugh”, from the self-enhancing subscale: “If something is difficult, it helps to find something funny about it”, from the aggressive subscale: “When I think of something that is funny about someone, I say it, even if it gets me into trouble” and the self-defeating humor subscale: “I often find myself laughing with others about things I am not very good at”. All items required a self-report tick box response on a Likert-type scale. The response scale scores were 1 = “Not at all like me”, 2 = “Not like me”, 3 = “A bit like me”, 4 = “A lot like me”. Subscale scores ranged from 6 to 24, with 6 being low for each humor scale and 24 being high for each humor scale. A mean score was then calculated for participants on each of the four humor subscales.

The Thinking and Feeling Questionnaire was selected as it overcomes the compartmentalization of different components of empathy and sympathy. It examined cognitive empathy, affective empathy and sympathy in parallel on separate subscales. The Thinking and Feeling Questionnaire was considered appropriate for the sample with a Flesch reading ease score of 85.40 (US grade level = 4.7, UK year 5, age 9-10 years). This measure presented children with 28 questionnaire items with each of the three dimensions measured on a sub-scale. One example from the affective empathy subscale is: “It makes me sad to see a child who can’t find anybody else to play with”, the cognitive empathy subscale: “I notice straight away when something makes my best friend unhappy” and the sympathy subscale: “I feel sorry for people who don’t have the things that I have”. Although Zoll and Enz (2005) supported the utilization of the Thinking and Feeling Questionnaire for children 8 years old and above, the decision was made to adapt the response scale for the present research. This research implemented a four-point response scale in preference of the original five-point response. The mid-point response was removed to avoid the potential tendency for children to opt for the neutral mid-point response (Borgers, Hox, & Sikkel, 2004). Thus, within the present research children responded to the Thinking and Feeling Questionnaire items on a Likert scale where response scale scores were 1 = “Strongly disagree”, 2 = “Disagree”, 3 = “Agree”, 4 = “Strongly agree”. Subscale scores varied as there were a different number of items on each. The cognitive empathy subscale had 12 items, with a score of 12 being low for that subscale and 48 being high. The affective empathy subscale had 10 items, where a score of 10 was low for that subscale and 40 was high. The sympathy subscale had 6 items, where a score of 6 was low for that subscale and 24 was high. One item on the sympathy subscale was reverse coded; “I don’t feel sorry for other children who are being picked on” as it was negatively worded. A mean score was calculated for each participant on each of the subscales.

### Procedure

Ethical approval was gained from the School of Psychology Ethics Committee. The head teachers from two primary schools were approached by email and agreement to participate was received following a discussion of the research. Parental consent letters that contained the details of the research were distributed by the school. The opt-out method was deemed appropriate as the research was not considered sensitive in nature. The researcher obtained any consent forms returned to the school and the class teacher ensured that those children (6 pupils) were not within the classroom at the time of data collection.

The researcher led the data collection sessions, which took place on a whole class basis. First, a standardized preamble was used to ensure that the instructions given were consistent. It was stressed to children that participation was their choice and despite their parents having given permission for them to take part: they could withdraw themselves at any point. Children were provided with the opportunity to ask any questions but did not need any clarifications at this stage. Children responded to the HSQ-Y followed by the Thinking and Feeling Questionnaire. At the end of the session children were given a full debrief in which the aims of the research were explained, children were reassured that their answers would remain confidential and informed that they should talk to a teacher if they had any further questions.

### Data Analysis Strategy

IBM SPSS Statistics 24 was used to conduct the data analysis. Statistical analyses included means, ranges and standard deviations to describe participants and Cronbach’s alpha to evaluate the internal consistency of the subscales on the HSQ-Y and Thinking and Feeling Questionnaire. Prior to further data analysis, it was necessary to conduct preliminary screening to ensure that the assumptions about the data were satisfied.

Pearson’s Product-Moment Correlation Coefficient was selected to examine relationships between subscales as there were two variables (humor styles and empathy), both measured on a continuous scale. The assumption of linearity was checked by plotting each humor style against each measure of empathy. A visual inspection of the scatterplots revealed a linear relationship between all variables. However, when the scatterplots were checked for the presence of outliers, it was found that this assumption was violated in four cases. According to Field (2013), these outliers could be retained in the analyses as each case did not exceed the boundary of 3.29 and -3.29 standard deviations from the mean. Based on the assumptions check, it was deemed acceptable to proceed with the correlational analysis.

A 2 (gender) x 2 (year group) MANOVA was selected to investigate gender and year group differences in humor styles and empathy. The assumptions of homoscedasticity and linearity were checked via a matrix of scatterplots; it was found that the variance surrounding the regression line was fairly equal for all values and that the independent variables were linearly related. Mahalonoblis distances were obtained to check multivariate normality. This assumption was violated as the value exceeded the maximum threshold, however further investigation revealed that the critical value was exceeded in only three cases and it was not necessary to remove these from the sample. The assumption of univariate normality was checked through an inspection of histograms, detrended normal Q-Q plots and boxplots. Although the assumption appeared satisfied in the figures, the Kolomogorov-Smirnov statistic suggested a possible violation of normality. However, as Pallant (2013) states that such a violation is common in larger sample sizes, this was not considered an issue. To check for multicollinearity, Pearson’s correlation was run to check the strength of association between the dependent variables. This test revealed that the dependent variables were moderately correlated. Next, the assumption of homogeneity of variance-covariance matrices were checked with an inspection of Box’s test, this revealed a value of .02, indicating that this assumption had been met. As there were no values less than .05 in Levene’s Test, it was presumed that the assumption of equality of variance had been met. Based on the assumptions check, it was deemed acceptable to proceed with MANOVA.

## Results

### Reliability Analysis

The reliability of all subscales on both the HSQ-Y and The Thinking and Feeling Questionnaire were reviewed. All subscales were found to have acceptable reliability .70 and above, the level considered satisfactory by Pallant (2013). Table 1 presents the Cronbach’s alphas for each subscale.

**Table 1:** Cronbach’s Alphas and n for Individual Subscales

Subscales	α	*n*
Affiliative	.87	210
Aggressive	.84	211
Self-enhancing	.70	211
Self-defeating	.80	210
Affective empathy	.81	203
Cognitive empathy	.71	203
Sympathy	.80	211

*Note.* Discrepancies in sample size due to illegible or incomplete questionnaires, total sample size; *n* = 214.

### Correlational Analysis

Preliminary assumption checks were satisfied. The relationships between each humor style and each dimension of empathy were investigated using Pearson product-moment correlation coefficient. Affiliative humor and cognitive empathy (*r* = .31, *n =* 214, *p* < .001) and self-enhancing and cognitive empathy (*r* = .35, *n =* 214, *p* < .001), affective empathy (*r =* .31, *n =* 214, *p <* .001) and sympathy (*r =* .25, *n =* 214, *p* < .001) were significantly and positively correlated, partially supporting the hypotheses. Aggressive humor was significantly and negatively correlated with sympathy (*r* = -.36, *n =* 214, *p* < .001) and affective empathy (*r =* -.28, *n =* 214, *p* < .001), also partially supporting the hypotheses. The findings for self-defeating humor did not support the hypotheses as there were no significant correlations found.

### Intercorrelations

Table 2 presents the intercorrelations between all variables in the research. Self-defeating humor was positively correlated with all three other humor styles; aggressive, (*r* = .47, *n =* 214, *p* < .001), affiliative, (*r* = .33, *n =* 214, *p* < .001) and self-enhancing, (*r* = .20, *n =* 214, *p* = .004). Affiliative humor was positively correlated with self-enhancing humor only, (*r* = .46, *n =* 214, *p* < .001). The remaining intercorrelations were non-significant. It was found that all measures of empathy were positively correlated with one another. The strongest association was between affective empathy and sympathy, (*r* = .72, *n =* 214, *p* < .001), next cognitive and affective empathy, (*r =* .44, *n =* 214, *p* < .001), sympathy and cognitive empathy, (*r* = .41, *n =* 214, *p* < .001).

**Table 2:** Intercorrelations Between Measures

Measures	1	2	3	4	5	6	7
1. Aff	-	.10	.46*	.33*	.12	.31*	.13
2. Agg		-	.04	.47*	-.28*	-.11	-.36*
3. SEn			-	.20*	.31*	.35*	.25*
4. SDf				-	-.03	.12	-.08
5. AffEmp					-	.44*	.72*
6. CogEmp						-	.41*
7. Symp							-

*Note.* Aff = affiliative; Agg = aggressive; SEn = self-enhancing; SDf = self-defeating; AffEmp = affective empathy; CogEmp = cognitive empathy; Symp = sympathy.

*p < .001.

### Gender and Year Group Differences

Preliminary assumption checks were satisfied. A 2 (gender) x 2 (year group) fully unrelated MANOVA was performed to investigate gender and year group differences in humor styles and empathy. This MANOVA accounted for all 7 dependent variables alongside gender and year groups in one analysis. See Table 3 for means and *SD*s. Analyses revealed a significant main effect of gender on aggressive humor, *F*(1, 208) = 7.24, *p =* .008, $\eta_{p}^{2}$ = .03 with boys reporting this humor style significantly more than girls: and a significant main effect of gender on affective empathy *F*(1, 208) = 30.81, *p* < .001, $\eta_{p}^{2}$ = .13 with girls reporting this type of empathy significantly more than boys. There was also a significant main effect of year group on self-enhancing humor, *F*(1, 208) = 30.81, *p* < .001, $\eta_{p}^{2}$ = .13 and self-defeating humor *F*(1, 208) = 13.19, *p* < .001, $\eta_{p}^{2}$ = .06, with increased reports of these humor styles among year 6 pupils and of year group on cognitive empathy *F*(1, 208) = 7.14 *p* = .008, $\eta_{p}^{2}$ = .03 with increased reports of these humor styles among year 6 pupils compared to year 5 pupils. There were no significant interaction effects.

**Table 3:** Means (and SDs) for Gender and Year Group Differences of Humor Styles and Empathy

Measures	Year 5	Year 6	Overall
Aff Male	3.20 (0.63)	3.23 (0.64)	3.21 (0.63)
Aff Female	3.22 (0.78)	3.25 (0.60)	3.23 (0.70)
Aff Overall	3.21 (0.71)	3.24 (0.61)	3.23 (0.67)
Agg Male	1.92 (0.68)	2.05 (0.64)	1.98 (0.66)
Agg Female	1.64 (0.67)	1.83 (0.64)	1.73 (0.66)
Agg Overall	1.77 (0.69)	1.93 (0.64)	1.84 (0.67)
SEn Male	2.50 (0.57)	2.98 (0.66)	2.73 (0.66)
SEn Female	2.52 (0.60)	2.97 (0.58)	2.73 (0.63)
SEn Overall	2.51 (0.59)	2.97 (0.61)	2.73 (0.64)
SDf Male	2.24 (0.69)	2.71 (0.76)	2.47 (0.76)
SDf Female	2.17 (0.72)	2.41 (0.68)	2.29 (0.71)
SDf Overall	2.20 (0.70)	2.55 (0.73)	2.37 (0.74)
AffEmp Male	2.90 (0.59)	3.03 (0.53)	2.96 (0.56)
AffEmp Female	3.22 (0.45)	3.34 (0.46)	3.28 (0.46)
AffEmp Overall	3.08 (0.54)	3.20 (0.51)	3.14 (0.53)
CogEmp Male	3.03 (0.41)	3.19 (0.39)	3.11 (0.40)
CogEmp Female	3.13 (0.39)	3.26 (0.36)	3.19 (0.38)
CogEmp Overall	3.09 (0.40)	3.23 (0.37)	3.15 (0.39)
Symp Male	3.35 (0.61)	3.52 (0.47)	3.44 (0.55)
Symp Female	3.50 (0.57)	3.60 (0.35)	3.54 (0.48)
Symp Overall	3.42 (0.58)	3.57 (0.41)	3.49 (0.51)

*Note.* 96 males, 116 females (year 5: n = 110, year 6: n = 102). Aff = affiliative; Agg = aggressive; SEn = self-enhancing; SDf = self-defeating; AffEmp = affective empathy; CogEmp = cognitive empathy; Symp = sympathy.

## Discussion

The present research was the first to address the association between children’s humor style use, empathy and sympathy. Although a handful of other researchers have conducted similar studies, these studies have focused on young adults and have employed the IRI, a measure which has been criticized for equating sympathy with empathy. Thus, the present research focused on childhood associations between humor styles, affective empathy, cognitive empathy and sympathy using the Thinking and Feeling Questionnaire which examined these constructs in parallel. The findings were generally supportive of the hypotheses and the previous literature, though it appeared that the associations between the variables are not as evident as previously assumed. This research indicated that different elements of empathy and sympathy are associated with different humor styles.

### Humor Styles and Empathy

The hypothesis that adaptive humor styles would be positively correlated with both measures of empathy and sympathy and that maladaptive humor styles would be negatively correlated with both measures of empathy and sympathy was partially supported, the results did not indicate such a simple association. Instead, different humor styles were related to specific components of cognitive empathy, affective empathy and sympathy.

Self-enhancing humor was the only style found to be positively correlated to both measures of empathy and sympathy. This suggests that children who use self-enhancing humor are able to conceptualize the perspective of another (cognitive empathy), understand their emotional condition (affective empathy) and sympathize with them. This finding contrasts with Hampes (2010) who observed a positive association between self-enhancing humor and perspective taking (IRI; Davis, 1980) empathy only. While Hampes (2010) suggested that self-enhancing is specifically associated with the perspective-taking elements of the cognitive empathy construct, in line with the intrapersonal function of this humor style, the findings from the present research suggest a more explicit link with interpersonal functioning. Perhaps there is some element of self-enhancing humor that is other orientated; as children learn to engage in prosocial behaviours, they are able to refine their empathic abilities and practice using adaptive styles of humor. Through interpersonal development, empathic abilities and use of self-enhancing humor could emerge together.

The link between self-enhancing humor and interpersonal functioning is further supported by the positive correlation with sympathy. Sympathy is an interpersonal characteristic, with early work on the topic describing it as the epitome of prosocial behaviour (Murphy, 1937). Perhaps children who have learnt to use self-enhancing humor to overcome adversity are able to transfer these skills into sympathetic action in an attempt to alleviate others’ emotional distress. This idea was supported by the observed intercorrelations between self-enhancing and affiliative humor in the present research. In addition to evidence that children who score highly on measures of empathy also score highly on measures of other altruistic behaviours, such as motivation to help others (Malti, Gummerum, Keller, & Buchmann, 2009). Thus, self-enhancing humor appears to include interpersonal elements, in addition to intrapersonal functions.

The findings for affiliative humor partially supported the hypothesis as it was found that this humor style was positively correlated with the cognitive construct of empathy only. These findings directly oppose Hampes (2010) who found that affiliative humor was instead solely associated with empathic concern, the affective component amongst young adults. It is somewhat surprising that the same correlation was not observed in the present research; after all, high levels of affiliative humor would be compatible with other prosocial behaviours, such as the ability to feel the emotions of others. This, therefore, might indicate that affiliative humor is significantly related to each component of empathy at different stages in development. For instance, cognitive empathy may be more significant amongst children as the cognitive capabilities to emotionally understand how others might perceive humor is still in development. It is only by age 9 that children are able to distinguish others’ emotions and understand subtle facial expressions (Gosselin, Perron, Legault, & Campenella, 2002). Perhaps the positive association between affiliative humor and cognitive empathy arises from the flourishing cognitive capabilities of the children in the present research. Alternatively, these findings may reflect the impact of Anderson’s (2014) recommendations regarding curriculum changes in the teaching of interpersonal skills in the UK. Perhaps the increased teaching of empathy and empathic skills, such as perspective-taking amongst junior-school aged children contributed to the differential outcomes from Hampes (2010). Overall, the findings for affiliative humor appear in line with those for self-enhancing humor; with the prosocial values of adaptive humor styles and empathy, children are able to understand how to use humor positively.

Aggressive humor was negatively correlated to affective empathy and sympathy. Although the correlational design does not permit an indication of the causal relationship, it is likely, based on previous literature that increased aggressive humor is associated with decreased affective empathy and sympathy. Hampes (2010) provided support for the negative association between aggressive humor and the affective components of empathy; individuals who have difficulty experiencing the emotional condition of others tend to use more aggressive humor. Perhaps in the present research, children who frequently use aggressive humor are not able to emotionally experience the feelings of others and so, do not understand the need to adapt their humor style to achieve more prosocial outcomes. This affective deficit may actually inhibit children’s development of adaptive humor styles as they enter a cycle in which aggressive humor limits social opportunities and limited social opportunities prohibit the opportunity to practice adaptive humor (Fox, Hunter, & Jones, 2016).

As aggressive humor was not significantly correlated with cognitive empathy, this might suggest that this humor style is related to an emotional deficiency, rather than a cognitive one. Perhaps children do possess the cognitive capabilities to engage in perspective-taking behaviours but are unable to use more adaptive humor styles as they lack the emotional capacity to appreciate how others perceive their use of humor. This mirrors the work of Klein and Kuiper (2006) who suggested that direct bullies’ use of aggressive humor reflected a deficiency of social and emotional skills. These bullies use aggressive humor to victimize their peers, though as they cannot feel the emotional impact that their humor has on others, they experience peer rejection.

Moreover, this affective deficit amongst those who use aggressive humor is likely to preclude sympathy. Children who are unable to feel the emotional distress of another through affective empathy are unlikely to experience the emotional drive to assist and alleviate distress. As sympathy has not previously been examined in relation to humor styles, there is no evidence to support this proposal. Though empirical evidence from interventions that target children’s interpersonal helping behaviours may provide some insight; Domitrovich, Cortes, and Greenberg (2007) found that interventions targeting children’s sympathetic responses led to a decrease in hostile interactions, arguments with peers and expressions of aggression.

Finally, self-defeating humor was not correlated to any of the measures of empathy. This absence of association reflects Hampes (2010) who also found non-significant relations between these variables. Perhaps one explanation of this is that self-defeating humor is self-orientated whilst empathy is predominantly other-orientated. Although self-orientated humor has been noted to affect others, for example resulting in lower quality relationships (Perry & Bussey, 1984), it appears that this might be an indirect effect that is not related to children’s perception and understanding of what others will find funny. It appears that variables other than empathy could explain the maladaptive nature and outcomes of this humor style. Additionally, the present results indicate that children are unable to self-report on self-directed humor styles, as James and Fox (2016a) suggested, some but not all children use and understand self-defeating humor at this age.

Overall, the findings from this research indicated that each humor style is differentially related to cognitive empathy, affective empathy and sympathy. It appears that these differential relationships were not dependent on the adaptive-maladaptive or self-other orientations of humor style, rather the different components of empathy and sympathy. Furthermore, these findings sustained the argument that cognitive empathy, affective empathy and sympathy are different constructs that should be measured separately in relation to other phenomena. Although these findings build on previous literature, more still needs to be done to examine why certain relationships might exist between different humor styles, the two components of empathy and sympathy in childhood but not later in development.

### Gender and Year Group Differences

There were several main effects of gender and year group for humor styles and empathy. Although these main effects partially supported the hypotheses, the effects observed were not as simple as initially hypothesized.

Notably, there was a significant main effect of gender on aggressive humor, with boys self-reporting this humor style more frequently than girls. This is in line with Martin et al. (2003) who found that male undergraduate students reported a much greater tendency to engage in aggressive expressions of humor, such as sarcasm and ridicule than females. As well as James and Fox (2016b) who found that boys aged 8-11 years old use maladaptive humor styles more frequently. Though contrary to the hypotheses, the same effect of gender was not found for the other maladaptive humor style, self-defeating humor. This is in contrast to Wu, Lin, and Chen (2016) who found that gender was predictive of the maladaptive humor styles, with males using both more frequently than females. The discrepancies between these findings might indicate cross-cultural gender differences between the samples. The relationships between humor styles and gender have been found to vary with cultural differences, across cultural influences and social norms (Martin, 2007). Perhaps it is these cultural differences that lead males from other cultures, such as Taiwan (Wu, Lin, & Chen, 2016), Belgium (Saroglou & Scariot, 2002) and Lebanon (Kazarian & Martin, 2004) to report self-defeating humor more frequently than those in the present research. Alternatively, it is also possible that gender differences in the use of self-defeating humor emerge later in life as each of the aforementioned studies were conducted with older samples.

There was also a main effect of gender on empathy, as boys self-reported affective component significantly less than girls. This finding is supportive of the generally accepted view that boys are less likely to engage in and exhibit empathy behaviours from the age of 4 and across the lifespan (Christov-Moore et al., 2014). Specifically males are also less likely to engage in emotionally orientated or prosocial behaviours (Roberts & Strayer, 1996) and report consistently lower scores on measures of affective empathy (Jolliffe & Farrington, 2006).

Overall, the effect of gender on humor styles and empathy was more complex than anticipated. The relationships found in the present research were more varied than those of Wu, Lin, and Chen (2016) who found that gender differences in all measures of empathy mediated gender differences across all styles of humor. It should be considered that developmental and cultural differences between Wu, Lin, and Chen’s (2016) Taiwanese sample of adolescents and that of the present research may reflect the observed discrepancies. The same effects of gender in humor styles and empathy were not found among English junior-school aged children.

### Limitations and Future Research

Although the present research made important contributions regarding the link between humor styles and empathy among children, it is not without its limitations. Firstly, the research is correlational and does not reveal causal relationships between particular humor styles and types of empathy. Although correlational research was appropriate as this study was the first to explore these variables among children, future research should seek to examine causal relationships. Moreover, future research should attempt to address the differential relationships between different styles of humor and different measures of empathy. It would be beneficial for future research to replicate the current findings and explore why, for example, affiliative humor is related only to cognitive empathy, but not affective empathy or sympathy. The divergence of this finding from associations found between affiliative humor and affective empathy among young adults should also be explored, with the intricacies between each humor style, empathy and sympathy examined across the lifespan and differing cultures.

### Evaluating the HSQ-Y

The HSQ-Y is a relatively new measure that appears to have good psychometric properties when utilized alongside measures of psychological wellbeing and adjustment (James & Fox, 2016b; James & Fox, 2018). However, the present research was the first to use the HSQ-Y alongside a measure of empathy in children. It was found that each of the subscales demonstrated adequate internal consistency, suggesting that the HSQ-Y is a reliable measure of the four humor styles among junior-school aged children.

However, the original HSQ has been challenged. For instance, Ruch and Heintz (2017) found that both humorous and non-humorous contexts could determine the HSQ scales, thereby suggesting that humor may not be the primary source of the variance within the HSQ scales, limiting their construct validity. Furthermore, that the relationships between the HSQ scales, the Big Five Personality traits and subjective wellbeing were reduced when non-humorous context within HSQ items were controlled for. Ruch and Heintz called for a reevaluation of the role of humor within the HSQ scales and their relationships to personality and wellbeing. Nevertheless, the evidence against the HSQ does not yet appear sufficient enough to dismiss the humor styles approach or its application to children.

### Conclusion

The present research has extended the humor styles literature with regard to younger children, magnifying the differential relationships of humor styles with cognitive empathy, affective empathy and sympathy. Future research should now aim to expand the foundation of knowledge surrounding these relationships in childhood, for example, considering the divergence of the associations between humor styles and empathy from those previously found among young adults. Moreover, the causal relationships between these variables should also be explored. In summary, this research has contributed a broader base of knowledge in the emerging area of children’s humor styles, opening the doors to the exploration of these relative to empathy and sympathy.
